# Antenatal health education with early postnatal booking and postnatal clinic attendance in primary health care centers in Bahrain: a quasi-experimental study

**DOI:** 10.3389/fpubh.2026.1938634

**Published:** 2026-10-20

**Authors:** Maitham E. E. Alhaddad, Mahmood A. H. Hubail, Maryam M. A. Rashed, Isa B. S. Alnajjar, Tasneem K. S. Husain, Fatema A. AlAali, Hala Al Asoomi, Basheer Makarem, Sadok Chlif, Fatema Habbash, Amer Almarabheh, Afif Ben Salah, Mohamed Hany Shehata

**Affiliations:** 1 Clinical Master in Family Medicine Program, Department of Family and Community Medicine, College of Medicine and Health Sciences, Arabian Gulf University, Manama, Bahrain; 2 Primary Health Care Centers, Manama, Bahrain; 3 Institut Pasteur de Tunis, Tunis, Tunisia; 4 Department of Family and Community Medicine, College of Medicine and Health Sciences, Arabian Gulf University, Manama, Bahrain; 5 American Mission Hospital, Manama, Bahrain; 6 Department of Family Medicine, Faculty of Medicine, Capital University, Cairo, Egypt

**Keywords:** Bahrain, early booking, health education, maternal health, postnatal care, postnatal clinic attendance, primary health care, quasi-experimental study

## Abstract

**Introduction:**

Postnatal care detects and manages complications in the weeks after delivery, yet in 2019 only 29% of women attending antenatal clinics in Bahrain’s primary health care (PHC) centers went on to attend the postnatal visit. We asked whether an antenatal intervention combining a standardized health-education session with early postnatal booking was associated with better knowledge of and attitudes toward postnatal care, and with higher postnatal clinic attendance.

**Methods:**

In a non-randomized quasi-experimental study, pregnant women at 36 weeks of gestation or above were enrolled at four PHC centers (two intervention, two control) between 21 March and 20 June 2024. Intervention-group women received a midwife-delivered education session, QR-coded material, and an early postnatal appointment; controls received standard care. Knowledge and attitude were measured before and immediately after the session in the intervention arm and once, at enrolment, in the control arm. Attendance was ascertained from electronic mother-and-child health records in both arms. Groups were compared with *t*-tests, chi-square or Fisher’s exact tests, and logistic regression adjusted for baseline differences.

**Results:**

Of 193 women approached, 175 (81 intervention, 94 control) were enrolled. Within the intervention arm, knowledge rose from 2.16 to 8.48 of 9 and attitude from 25.79 to 35.89 of 40 (both *p* < 0.001); because the control arm was assessed only once, these within-group changes cannot be attributed to the intervention. Attendance was 72.7% in the intervention arm versus 41.3% in controls (odds ratio 3.79, 95% CI 1.98–7.27; adjusted odds ratio 5.55, 95% CI 2.04–15.08). Knowledge and attitude did not independently predict attendance. Attendance varied widely between the two control centers (26.5 and 58.1%), and the intraclass correlation of 0.091 implies an effective sample of about 36 women.

**Conclusion:**

Antenatal education with early postnatal booking was associated with substantially higher postnatal clinic attendance. With four non-randomized centers and between-center variation as large as the difference between arms, the design cannot separate an intervention effect from center-level differences. Early booking with structured counseling may be a feasible strategy for improving postnatal-care uptake and warrants evaluation in a cluster-randomized design.

## 1 Introduction

Postnatal care (PNC) is an integral component of global maternal and child health, yet post-birth and neonatal deaths remain a heavy burden worldwide (1). Timely identification of high-risk pregnancies and adequate postnatal care can prevent a substantial proportion of this burden among mothers and newborns (2–5). Postpartum depression, postpartum hypertension, and anemia—which typically peak between three and 6 days after childbirth—are among the significant health concerns women face after delivery (6–8), while exclusive breastfeeding and sexual-health counseling are further important components of postnatal care (9, 10).

Reported PNC utilization varies widely, from 67.9 to 95.1% in some countries (11–14) to between 5.8 and 45% in others, although it is rising in several settings (15–22). Bahrain sits toward the lower end of that range, for reasons set out below.

Factors associated with PNC uptake have been described in many settings and include sociodemographic, cultural, and economic characteristics, knowledge and awareness, delivery complications, and health-seeking behavior (23–35). The interconnectedness of early antenatal care, facility delivery, and early postnatal care underscores the role of health-care workers in ensuring continuous, integrated care (36), and a mixed-method study of postnatal care in rural China found that staff shortages limited the ability of maternal and child health workers to reach women at home, highlighting the value of in-service training and supervision (37).

Cultural and religious barriers, together with limited awareness, contribute to low routine care-seeking and a preference for traditional healers over skilled providers (38–40). Asim et al. identified three sociocultural barriers in Pakistan: aversion to biomedical interventions, gendered imbalances in decision-making, and restrictions on women’s mobility (41). Cultural beliefs are closely intertwined with socioeconomic status, education, and empowerment, which further influence maternal health-service utilization (42, 43). Improving the availability and accessibility of antenatal care can have a cascading effect, enhancing facility delivery and ultimately PNC utilization (44); similarly, birth order, maternal education, and awareness of facilities with skilled professionals consistently predict the use of skilled antenatal and delivery care (45). Difficult terrain and a lack of transport infrastructure hinder access in some settings, such as the Debre Libanos District of Ethiopia (46), and geographic accessibility and waiting time during PNC are reported barriers elsewhere (47, 48).

Four strands of intervention evidence bear directly on the present study. The first is provider-delivered antenatal counseling about postnatal care. Gitonga et al. observed that routine health education delivered by trained providers, coupled with greater involvement of community health workers, can enhance PNC uptake (49), and Shiferaw et al., in a prospective controlled study in Ethiopia, showed that combining an intervention with provider training in client-centered care increased the proportion of women who, having received antenatal care, also used postnatal care at the same center (50); this is the closest published analogue to the pathway tested here. The second is appointment support and reminders, which have mostly been studied in digital form: a 2023 systematic review and meta-analysis by Gayesa et al. found that mobile-health interventions improved institutional delivery, PNC utilization, exclusive breastfeeding, and knowledge of obstetric danger signs in low- and lower-middle-income countries (51), and a locally developed mHealth intervention improved uptake of recommended postnatal services in Nigeria (52). The third is multicomponent programs: a pre/post evaluation of multifaceted strategies in an Australian hospital improved women’s perceptions of the quality of postnatal care (53), a Swedish quality-improvement program improved maternal satisfaction across successive cohorts (54), and community-based intervention packages have substantially increased early breastfeeding rates (55). The fourth is evidence from primary-care and comparable service settings, including work on the quality of and satisfaction with postnatal services in Saudi Arabia (56), videoconferencing-based postpartum care in Catalonia, which reduced health-center visits while enabling timely advice (57), and web-based postnatal psychoeducation for first-time mothers, which improved self-efficacy and reduced postnatal depression (58).

What this literature does not contain is an evaluation of advance booking of the postnatal consultation during antenatal care. Interventions have concentrated on informing women or on reaching them after discharge, rather than on removing the organizational step that a woman must complete herself once she has delivered. Nor has the combination of provider-led counseling with a concrete appointment been tested in a Gulf primary-care system, where postnatal care is free at the point of use and cost is therefore unlikely to be the operative barrier. Local circumstances also shape how such an intervention should be designed: comorbidity is common in this population (59), and service delivery must remain robust to disruption, as the rapid reorganization of care during the COVID-19 pandemic showed (60).

Against this background, the situation in Bahrain has features of its own. Postnatal care is provided free of charge in all PHC centers as a single visit booked between 6 weeks and 6 months after delivery. It aims to detect late postpartum complications, early complications being managed in secondary care, and to provide a general assessment of the mother’s health, screening for postnatal depression, and counseling on breastfeeding and family planning (1, 2, 9, 10). Service statistics compiled by the Primary Health Care Directorate for 2019 showed that only 29% of women who attended antenatal clinics went on to attend the postnatal visit (unpublished data, Primary Health Care Directorate, Ministry of Health, Kingdom of Bahrain); 2019 is used as the reference year because postnatal services moved to teleconsultation during the COVID-19 pandemic in 2020 and 2021. The first national study of why attendance is so low, a cross-sectional survey of 319 mothers attending PHC centers in January 2023, found that 31% were unaware the clinic existed, 42% were aware but had not attended, and only 27% had attended (61). Attendance was associated with having been counseled by a provider about the visit, its content, and how to book it; the leading reasons for staying away were childcare responsibilities (41%), a belief that the visit was unimportant (38%), reluctance to undergo a pelvic examination (37%), and not knowing how to book (25%) (61). Because the barriers were largely informational and organizational, the authors recommended embedding postnatal counseling in third-trimester antenatal care and simplifying booking (61). Those are the two components tested here, in agreement with the Primary Health Care Directorate.

The aim of this study was to provide evidence to primary-care decision-makers to improve the uptake of postnatal consultations in Bahrain. The specific objectives were (i) to measure the impact of an intervention combining health education and early booking on women’s knowledge of and attitudes toward postnatal consultations, and (ii) to measure its impact on the uptake of postnatal consultations.

## 2 Materials and methods

### 2.1 Study design

The study was conducted as the quality-improvement project of the Clinical Master in Family Medicine Program of the College of Medicine and Health Sciences, Arabian Gulf University, Manama, Bahrain, by candidates enrolled in the program and under the supervision of faculty of the Department of Family and Community Medicine of the same college, in collaboration with the Primary Health Care Directorate, Ministry of Health, Kingdom of Bahrain. We conducted a two-arm, non-randomized, quasi-experimental study in four primary health care centers in the Kingdom of Bahrain, two serving as intervention centers and two as controls. Allocation was at the level of the center rather than the individual woman and was not random; how the centers were selected and assigned is described in Section 2.2. In the intervention centers, women received a health-education session and an early booking for the postnatal consultation during antenatal care at 36 weeks of gestation or above, and knowledge and attitude were assessed immediately before and after the session. In the control centers the same scales were administered once, at enrolment, with no later reassessment; the consequences of this for the analysis are set out in Sections 2.9 and 4.6. Postnatal clinic attendance, the behavioral outcome, was ascertained identically in both arms.

### 2.2 Study population and sample

Postnatal services in Bahraini PHC centers are provided free of charge to Bahraini women and to non-Bahraini women married to Bahrainis. Eligible participants were pregnant women at 36 weeks of gestation or above attending the antenatal clinic in the selected centers who met the inclusion criteria and provided written informed consent. Non-Bahraini women not married to Bahrainis were excluded to avoid incomplete follow-up of services for which they are not eligible. The four participating centers were chosen by convenience sampling by the mother-and-child health (MCH) management of the Primary Health Care Directorate, not by the investigators, and before the intervention materials were developed. Three criteria were applied: the flow of patients through the center, the availability of antenatal and postnatal services on most days of the week, and the presence of nurse-midwives already trained in health education. All three concern the practicability of running the study in a given center and were applied to the selection of the four centers as a group; they do not describe how the four were subsequently divided between the arms, and the basis of that division was not documented. Allocation of the four centers to the intervention and control arms was not random. This is therefore a non-randomized controlled quasi-experimental study, and the possibility of selection bias at the level of the center cannot be excluded. The two intervention centers lie in the Southern Governorate and the two control centers in the Northern Governorate, so the arms are geographically separate and serve distinct catchment populations. Separation of this kind makes contamination between arms unlikely, since women and midwives in one arm had no routine contact with the other, but it also means that study arm is completely confounded with governorate. Any systematic difference between the two governorates in service organization, catchment population, or historical postnatal attendance would be indistinguishable from an effect of the intervention. The centers were chosen to be similar in the population served, the composition of health professionals, the organization of care, and the frequency of weekly antenatal and postnatal consultations, but this similarity was a selection criterion, not a demonstrated equivalence: we did not obtain center-level data on service volume, staffing establishment, or pre-study postnatal attendance, and we are therefore unable to demonstrate baseline comparability at the level of the center. The observed participant-level differences in age and family income between arms (Table 1) indicate that the catchment populations were not in fact equivalent.

**Table 1:** Sociodemographic characteristics of participants by study group (*N* = 175).

Variable		Control *n* (%)	Intervention *n* (%)	*p*-value
Age	<25 years	22 (23.4)	9 (11.1)	0.007
	25–30 years	44 (46.8)	30 (37.0)	
	>30 years	28 (29.8)	42 (51.9)	
Education	Intermediate or below	13 (13.8)	13 (16.0)	0.856
	Secondary	31 (33.0)	24 (29.6)	
	College or above	50 (53.2)	44 (54.3)	
Employment	Unemployed	61 (64.9)	50 (61.7)	0.405
	Student	7 (7.4)	3 (3.7)	
	Employed	26 (27.7)	28 (34.6)	
Family income	<500 BD	38 (40.4)	10 (12.3)	<0.001
	500–1,000 BD	47 (50.0)	58 (71.6)	
	>1,000 BD	9 (9.6)	13 (16.0)	
Husband education	Intermediate or below	15 (16.0)	7 (8.6)	0.163
	Secondary	42 (44.7)	32 (39.5)	
	College or above	37 (39.4)	42 (51.9)	

Values are *n* (%). Percentages are calculated within each study group (control *n* = 94; intervention *n* = 81), not on the total sample. *p*-values are from Pearson’s chi-square test. BD, Bahraini dinar. Significant baseline differences: age (*p* = 0.007) and family income (*p* < 0.001).

### 2.3 Sample size

The sample size was calculated for a one-sided test comparing two proportions—the expected proportion of postnatal attendance in the intervention group (P1 = 50%) and in the control group (P2 = 30%)—following the method of Fleiss (62), with a 5% significance level and 80% power at a 1:1 allocation ratio. This yielded 83 participants per group; allowing for 20% loss to follow-up or non-response, the target was 100 per group (200 in total). For the subsequent statistical analyses, two-sided tests were used, with a *p*-value <0.05 considered statistically significant. The calculation assumed independent observations at the level of the individual woman and did not incorporate clustering or an intracluster correlation coefficient. Because the intervention was allocated at the level of the center, the effective sample size is smaller than the number of women enrolled, and the study was not powered for a cluster-level causal comparison. With two centers per arm, adequate power for such a comparison could not have been achieved by enrolling more women; it would require a substantially larger number of centers. The sample size reported here should therefore be read as supporting an individual-level comparison of women attending the two groups of centers, not as a basis for a cluster-level causal estimate.

### 2.4 Inclusion and exclusion criteria

Women were eligible if they were Bahraini or non-Bahraini married to a Bahraini, were attending the antenatal clinic at 36 weeks of gestation or above, were older than 18 years, were willing to participate and provided consent, and used a smartphone. Women were excluded if they did not speak Arabic or English, were at high risk in the current pregnancy (diabetes mellitus, hypertension, or sickle cell disease), or were unable to understand the educational material. Smartphone ownership was applied as an eligibility criterion in both arms rather than in the intervention arm alone. Restricting it to the intervention arm would have made the two groups non-comparable on a characteristic plausibly related to health literacy, digital access, and socioeconomic position, and would have confounded the comparison it was intended to serve. Applying it uniformly preserves comparability at the cost of generalizability, since women without a smartphone—who may face the greatest barriers to postnatal care—are not represented in either arm. This restriction is acknowledged in Section 4.6.

### 2.5 Centers and data collection

Four centers took part, two in the Northern Governorate and two in the Southern Governorate. The intervention centers were Yousef Abdulrahman Engineer Health Center (Isa Town) and Shaikh Abdulla Bin Khaled Al Khalifa Health Center (Riffa); the control centers were Kuwait Health Center (Karzakan) and Mohammed Jasim Kano Health Center (Hamad Town). Recruitment and baseline data collection took place on all working days during antenatal clinics between 21 March and 20 June 2024, during working hours (07:00–14:00), by six interviewers assigned to the different centers. The interviewers were not blinded to allocation, since the intervention was delivered in their own centers.

Postnatal clinic attendance was ascertained by passive review of the electronic mother-and-child health record at the woman’s own health center, identified by her national identification number. Participants were not contacted and no self-report was used. A woman was counted as having attended if a postnatal consultation was documented in that record, whether delivered by the physician or by the staff nurse of the mother-and-child health clinic, since the national program provides for either; the recommended interval is 6 weeks to 6 months after delivery. A visit made at a different primary health care center would not have been captured, and teleconsultation was not counted; none was recorded, postnatal services having returned to face-to-face delivery after the pandemic period. Records were reviewed in 2025, after the six-month window had closed for the last woman enrolled. The same record system, case definition, and review procedure were applied in both arms. For six women the index delivery could not be matched in the record, most often because the identification number recorded at enrolment could not be verified; their outcome is treated as missing, not as non-attendance.

### 2.6 Intervention

The intervention had three components, delivered together to every woman in the intervention arm; no woman received a subset. First, mother-and-child health (MCH) midwives were trained to deliver a standardized health-education session to women attending antenatal clinics at 36 weeks of gestation or above, covering the importance of the postnatal consultation, its benefits for mother and newborn, and the services provided. The session was delivered individually by the trained midwife during the routine antenatal visit, immediately before the post-intervention questionnaire, and followed fixed content. Second, women were given an early booking for a postnatal consultation between 6 weeks and 6 months after delivery, following a written standard operating procedure developed to standardize the booking process (Supplementary material). Third, the educational material was made available through a QR code for further reading. Because the three components were never varied independently, the design cannot attribute any difference in attendance to one of them. We did not record the duration of the session or of the midwife training, used no delivery checklist beyond the booking procedure, and did not monitor fidelity by observation or audit; consistency of delivery rests on the standardized content and booking procedure, not on measured adherence.

### 2.7 Control

Control-group women received the standard of care defined by the national postnatal care program. Under that standard, the mother-and-child health midwife tells the woman verbally, at some point during antenatal care, that a postnatal consultation exists and that she should arrange it herself after delivery. No structured education session is delivered, no written or QR-coded material is given, no appointment is made in advance, and no reminder is issued; providers in the control centers had no facility to book the consultation before delivery, since under the national program booking takes place afterward and at the woman’s own initiative. The contrast between the arms is therefore between an organized care pathway and an unassisted one, not between the presence and absence of information. We are not aware of any other postnatal-care initiative introduced in the participating centers during the study period.

### 2.8 Study instrument and variables

A structured, interviewer-administered questionnaire collected information on (i) identification; (ii) sociodemographic characteristics (age, education of the woman and her husband, employment status, marital status, and average monthly income); (iii) gynecological, obstetric, and medical history (previous deliveries, mode of delivery, whether the pregnancy was planned, mode of conception, comorbidities, daily medications, and previous complications during and after delivery); (iv) awareness, knowledge, and attitudes regarding postnatal visits; and (v) documentation of postnatal-visit attendance. It included two primary outcome measures: a 9-item knowledge scale, with dichotomous items summed to a total of 0 to 9, and an 8-item attitude scale, with five-point Likert items summed to a total of 8 to 40; higher scores are more favorable on both. Three of the eight attitude items (items 1, 3, and 7) are worded in the negative direction and their scores were reversed before the total was formed, an assignment confirmed by the item covariance as well as by the wording. Totals were computed as the unweighted sum with no imputation for missing items. The items were developed by the research team, drawing on the instrument used in the 2023 national survey (61) and on the World Health Organization recommendations on postnatal care; neither the source instrument nor the present one has been formally validated, and we make no claim to established construct validity. The questionnaire was developed in English, translated into Arabic, back-translated by an independent bilingual Bahraini with discrepancies reconciled, and piloted among women from centers not taking part in the study to assess relevance, feasibility, and acceptability. Internal consistency was assessed on the study sample. Because the knowledge items are dichotomous, the Kuder–Richardson formula 20 coefficient is reported for that scale (0.672 at baseline across all 175 participants; 0.932 after the education session in the intervention arm), and Cronbach’s alpha for the attitude scale (0.838 at baseline; 0.811 after the session). The lower baseline knowledge coefficient reflects the low and narrow distribution of scores before the session, when most women answered few items correctly.

In the intervention centers the questionnaire was administered twice in the same visit, immediately before and immediately after the education session. In the control centers it was administered once, at enrolment. There is therefore no follow-up measurement of knowledge or attitude in the control arm, and the pre/post comparison in Sections 3.5 and 3.6 describes change within the intervention arm only. Postnatal clinic attendance, by contrast, was ascertained identically in both arms and is a genuine between-group comparison.

### 2.9 Statistical analysis

Categorical variables are presented as frequencies and percentages and continuous variables as mean ± standard deviation (SD). Knowledge and attitude scores were compared before and after the education session in the intervention arm with paired-samples t-tests, and between arms at baseline with independent-samples *t*-tests, using the Welch correction where Levene’s test indicated unequal variances. Effect sizes are reported as Cohen’s *d*; for the paired comparisons the standardizer is the SD of the paired differences (*d*_z_). Because the control arm was assessed only once, no difference-in-differences estimate of change in knowledge or attitude could be computed, and the within-group comparison is reported descriptively, not as evidence of an intervention effect.

Attendance was compared between arms with the chi-square test, with the odds ratio as the effect measure; Fisher’s exact test was used wherever an expected cell count fell below five, and all exact *p*-values are two-sided. Knowledge and attitude were dichotomized at the sample mean of each scale at baseline (knowledge 1.74; attitude 30.26), giving cut-points of ≥ 2 for high knowledge and ≥ 31 for positive attitude; these are data-derived thresholds, not validated ones and were fixed before the regression was fitted. A binary logistic regression then estimated the association between the intervention and attendance adjusted for age, family income, knowledge, and attitude. The model was not prespecified: the covariates were chosen because they differed between arms at baseline, with age and income also established correlates of maternal service use. Selecting covariates on baseline *p*-values is not a reliable way to identify confounders, and we present the model on that understanding. Knowledge and attitude carry a further caution: the intervention was designed to change them, so they may lie on the causal pathway to attendance instead of confounding it, and conditioning on a mediator estimates a direct effect, not a total one. The unadjusted comparison is therefore the more appropriate estimate of the total association. Model calibration was assessed with the Hosmer–Lemeshow test, explained variation with Cox and Snell and Nagelkerke *R*^2^, and collinearity with variance inflation factors.

Allocation was at the level of the center, so observations within a center are not independent and individual-level tests will tend to understate standard errors. With two centers per arm, neither a mixed-effects model nor a cluster-robust variance estimator can be estimated with any stability, and the center cannot enter the adjusted model as a fixed effect because it is collinear with study arm; none of these was fitted. Instead we report attendance for each center separately (Table 2) and estimated the intraclass correlation coefficient for attendance within study arms, so that the intervention effect itself does not contribute to the between-center variance, together with the design effect 1 + (m − 1)ρ and the corresponding effective sample size, where m is the mean number of women per center. As a further sensitivity analysis the primary comparison was repeated under the four extreme assumptions about the six women whose attendance could not be ascertained. The analyses of obstetric and medical correlates (Section 3.9) and of the relationship between knowledge, attitude, and attendance (Section 3.10) were exploratory, were not adjusted for multiple comparisons, and are hypothesis-generating. All tests were two-sided with *p* < 0.05 taken as significant. Analyses were performed in IBM SPSS Statistics version 30 and the regression diagnostics, intraclass correlation, and sensitivity analyses were computed in Python 3 (statsmodels 0.15).

**Table 2:** Postnatal clinic attendance by study group and by individual health center.

Group	Attended *n* (%)	Did not attend *n* (%)	Total	*χ*^2^ (df)	*p*	OR (95% CI)
Intervention	56 (72.7)	21 (27.3)	77	16.77 (1)	<0.001	3.79 (1.98–7.27)
Control	38 (41.3)	54 (58.7)	92			
Intervention center 1	23 (69.7)	10 (30.3)	33			
Intervention center 2	33 (75.0)	11 (25.0)	44			
Control center 1	13 (26.5)	36 (73.5)	49			
Control center 2	25 (58.1)	18 (41.9)	43			
Total	94 (55.6)	75 (44.4)	169			

Attendance is shown for each of the four participating centers so that the consistency of the difference within each arm can be judged. The chi-square test, *p*-value and odds ratio refer to the comparison between study arms. Percentages are of the row total.

### 2.10 Ethical considerations

Ethical approval was obtained from the Research and Ethics Committee of the College of Medicine and Medical Sciences, Arabian Gulf University (reference number E34-PI-02-24, approved 10 March 2024), and from the Ethics Committee of Primary Health Care, Ministry of Health, Kingdom of Bahrain (serial number 30, approved 10 March 2024). Written informed consent was obtained from all participants before data collection. Participants were informed of the study’s purpose, assured of confidentiality and the absence of coercion, and interviewed in private.

## 3 Results

### 3.1 Sociodemographic characteristics

A total of 193 pregnant women at 36 weeks of gestation or above, attending an antenatal appointment in the selected PHC centers between 21 March and 20 June 2024, were approached and screened. Two women attending control centers were ineligible—one non-Bahraini not married to a Bahraini and one aged 17 years—and four records from the intervention centers were removed at data cleaning as duplicates or because the national identification number could not be verified. A further 12 women began the questionnaire but did not complete it, either declining to continue, discontinuing part-way, or proving untraceable in the electronic record system. This left 175 women who consented to and completed the study, 81 in the intervention group and 94 in the control group, a participation rate of 90.7% of those approached (Figure 1). Enrolment fell short of the planned 100 participants per group in both arms, and the intervention arm was two participants below the minimum calculated requirement of 83 per group; the implications are considered in Section 4.6.

**Figure 1:**
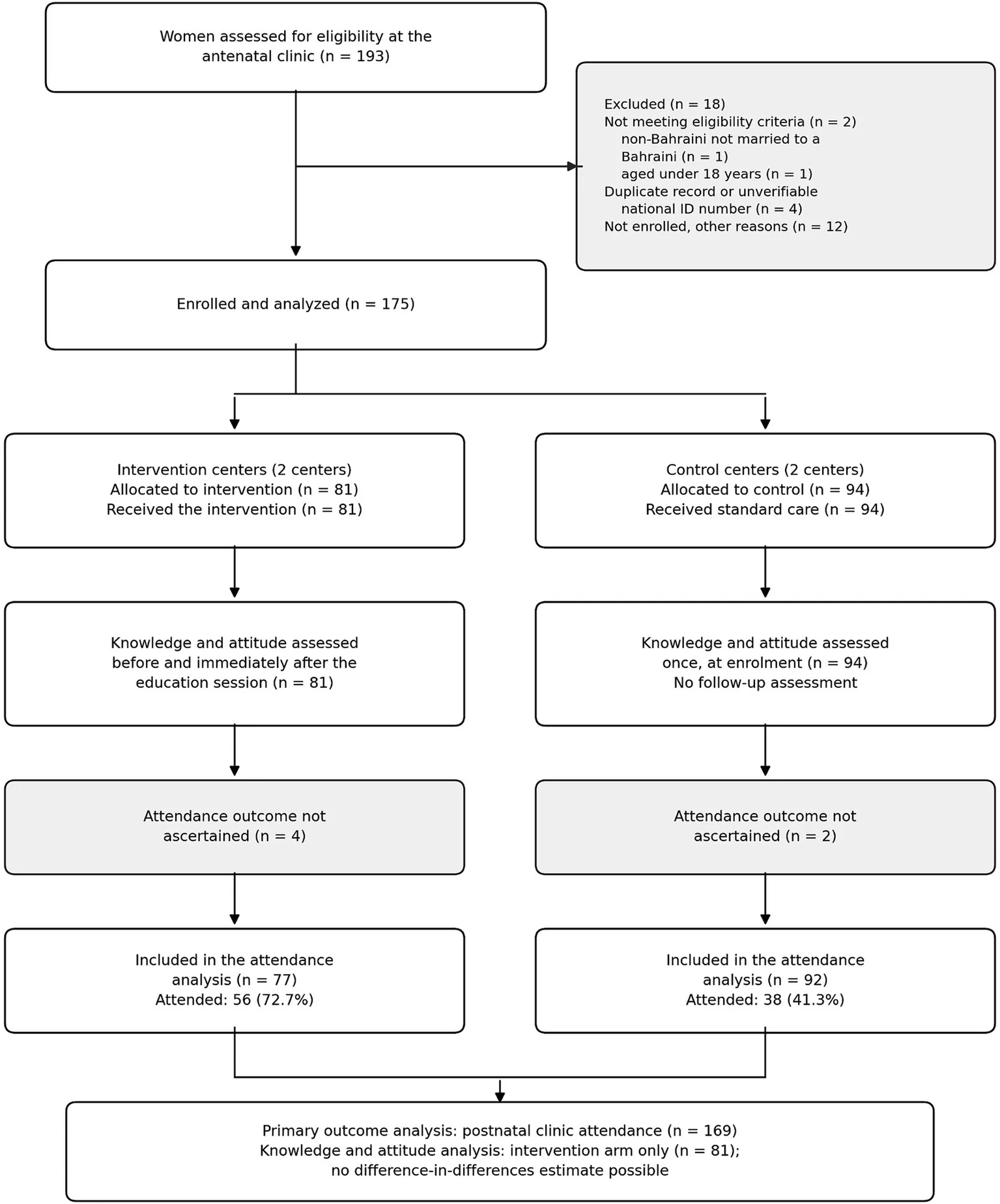
Participant flow through the study, from women approached at the antenatal clinic to those included in the attendance analysis.

In the control group, most participants were aged 25–30 years (44; 46.8%), followed by those older than 30 years (28; 29.8%) and those younger than 25 years (22; 23.4%). In the intervention group, the largest category was older than 30 years (42; 51.9%), followed by 25–30 years (30; 37.0%) and younger than 25 years (9; 11.1%). The groups differed significantly in age distribution (*p* = 0.007), the intervention group being older. College education or above was the most common educational level in both the control (50; 53.2%) and intervention (44; 54.3%) groups, with no significant difference (*p* = 0.856). Employment status was comparable (*p* = 0.405). Family monthly income differed significantly (*p* < 0.001): in the control group, 47 (50%) earned 500–1,000 BD, 38 (40.4%) below 500 BD, and 9 (9.6%) more than 1,000 BD, whereas in the intervention group, 58 (71.6%) earned 500–1,000 BD, 13 (16.0%) more than 1,000 BD, and only 10 (12.3%) below 500 BD, indicating a more affluent intervention group. The husband’s educational level was comparable between groups (*p* = 0.163) (Table 1).

### 3.2 Obstetric and medical characteristics

The two groups were comparable across all obstetric and medical characteristics, with no significant between-group differences for previous pregnancy (*p* = 0.114), mode of last delivery (*p* = 0.287), previous postnatal complications (*p* = 0.391), previous antenatal complications (*p* = 1.000), whether the current pregnancy was planned (*p* = 0.524), mode of conception (*p* = 0.500), diabetes mellitus (*p* = 1.000), sickle cell disease (*p* = 0.463), or use of daily medication (*p* = 0.281). Apart from age and socioeconomic indicators, the groups were therefore clinically comparable (Table 3).

**Table 3:** Obstetric and medical characteristics of participants by study group.

Variable		Control *n* (%)^a^	Intervention *n* (%)^a^	*p*-value
Previous pregnancy	No	18 (19.1)	24 (29.6)	0.114
	Yes	76 (80.9)	57 (70.4)	
Mode of last delivery: (control *n* = 76; intervention *n* = 57)^b^	Vaginal	73 (96.1)	52 (91.2)	0.287
	Cesarean	3 (3.9)	5 (8.8)	
Previous postnatal complications: (control *n* = 76; intervention *n* = 57)^b^	No	72 (94.7)	56 (98.2)	0.391
	Yes	4 (5.3)	1 (1.8)	
Previous antenatal complications (control *n* = 76; intervention *n* = 56)^b,c^	No	75 (98.7)	56 (100)	1.000
	Yes	1 (1.3)	0 (0.0)	
Current pregnancy planned (control *n* = 94; intervention *n* = 80)^c^	No	34 (36.2)	25 (31.3)	0.524
	Yes	60 (63.8)	55 (68.8)	
Mode of conception	Normal	92 (97.9)	81 (100)	0.500
	Induced	2 (2.1)	0 (0.0)	
Diabetes mellitus	No	93 (98.9)	81 (100)	1.000
	Yes	1 (1.1)	0 (0.0)	
Sickle cell disease	No	94 (100)	80 (98.8)	0.463
	Yes	0 (0.0)	1 (1.2)	
Daily medication	No	70 (74.5)	66 (81.5)	0.281
	Yes	24 (25.5)	15 (18.5)	

^a^Values are *n* (%). Percentages are calculated within each study group using the denominator shown for that variable. Variables applicable to all participants use the total number in the respective study group (control *n* = 94; intervention *n* = 81).

^b^Restricted to women reporting a previous pregnancy (control *n* = 76; intervention *n* = 57); the denominator for these variables is the number of eligible women in each group, and the eligible *n* is stated in the row label.

^c^Data were missing for one intervention-group participant on previous antenatal complications and for one on whether the current pregnancy was planned; percentages for these two variables use the number of participants with available data as the denominator. *p*-values are from Pearson’s chi-square test or Fisher’s exact test (two-sided) where any expected cell count was below five.

### 3.3 Baseline knowledge and attitude

Before the intervention, the groups differed significantly on both scales. Baseline knowledge was higher in the intervention group (2.16 ± 2.14) than in the control group (1.38 ± 1.34). Levene’s test indicated unequal variances for this scale (*F* = 6.87, *p* = 0.010), so the Welch correction was applied: *t*(130.4) = −2.83, *p* = 0.005 (Cohen’s *d* = 0.44). Baseline attitude was substantially higher in the control group (34.12 ± 4.25) than in the intervention group (25.79 ± 4.18), a difference of 8.33 points on the 8–40 scale; *t*(173) = 13.02, *p* < 0.001 (Cohen’s *d* = 1.97). This is a large imbalance and is discussed in Section 4.6. Together with the baseline differences in age and income, these imbalances justified the adjusted analysis presented in Section 3.8 (Table 4).

**Table 4:** Baseline (pre-intervention) knowledge and attitude scores by study group.

Scale (range)	Control mean ± SD	Intervention mean ± SD	*t* (df)	*p*-value	Cohen’s *d*
Knowledge (0–9)	1.38 ± 1.34	2.16 ± 2.14	−2.83 (130.4)	0.005	0.44
Attitude (8–40)	34.12 ± 4.25	25.79 ± 4.18	13.02 (173)	<0.001	1.97

Control *n* = 94; intervention *n* = 81. Two-sided independent-samples *t*-test.

### 3.4 Sources of awareness of the postnatal clinic

Among participants aware of the postnatal clinic, the most frequently reported sources of information were friends (20%) and family members (18%), followed by doctors during the antenatal visit (17%) and doctors after delivery (16%). The MCH clerk (12%), nurses or midwives (8%), and social media (7%) were cited less frequently, while only 3% reported learning about the clinic through a formal health-education session (Figure 2; multi-response question, *n* = 174). Informal social networks (friends and family, together 38%) were thus the leading source of awareness, whereas the structured channels best suited to accurate, standardized messaging—formal health-education sessions (3%) and social media (7%)—were the least used.

**Figure 2:**
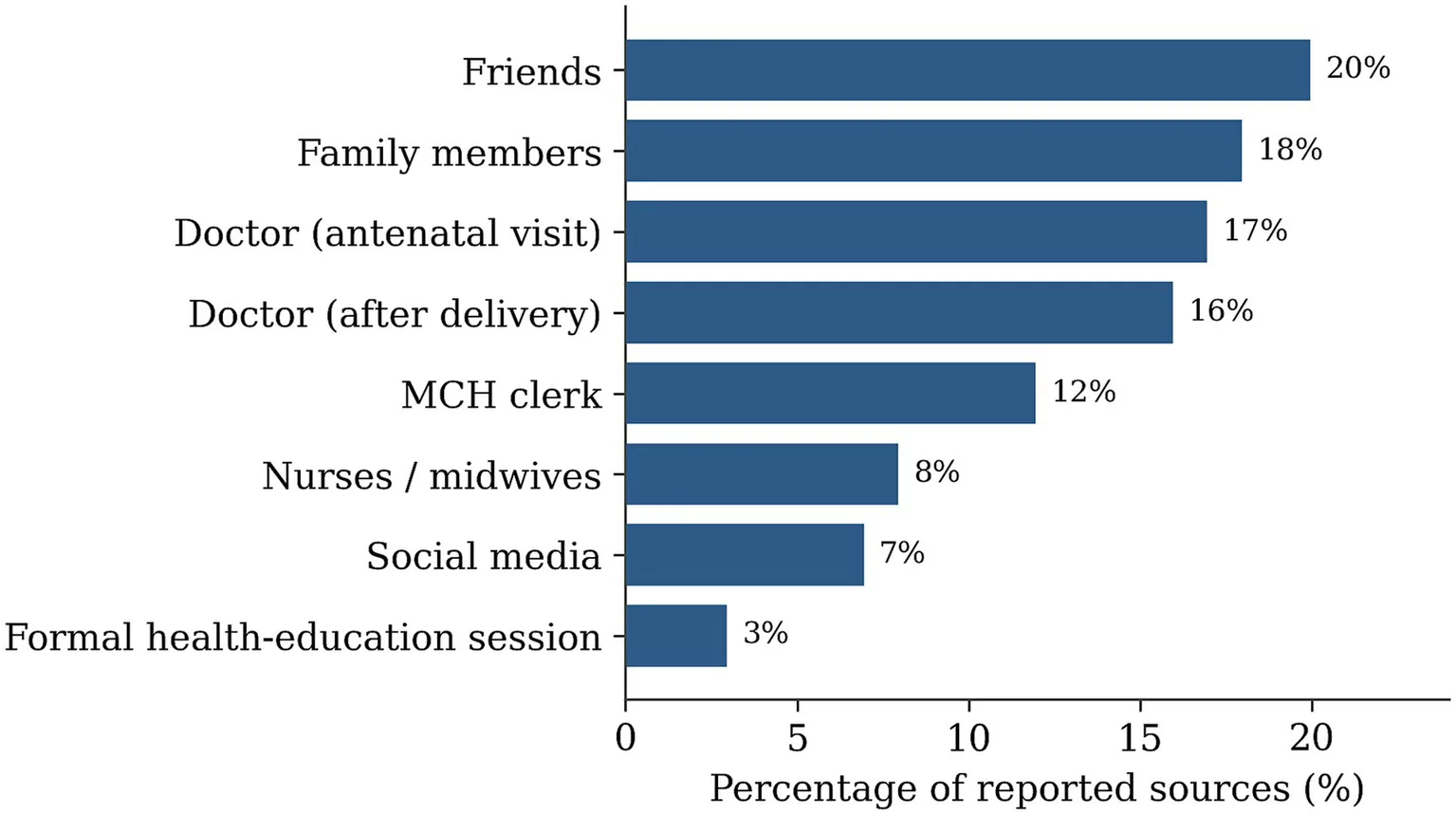
Reported sources of awareness of the postnatal clinic among participants aware of the service (multi-response; *n* = 174).

### 3.5 Impact of the intervention on knowledge

Within the intervention group, knowledge improved markedly after the educational session. The mean knowledge score rose from 2.16 ± 2.14 before the intervention to 8.48 ± 1.69 afterward, a mean increase of 6.32 points (95% CI 5.62–7.03). This change was highly significant on the paired-samples *t*-test, *t*(80) = 17.84, *p* < 0.001, with a very large effect size (Cohen’s *d*_z_ ≈ 1.98) (Figure 3; Table 5).

**Figure 3:**
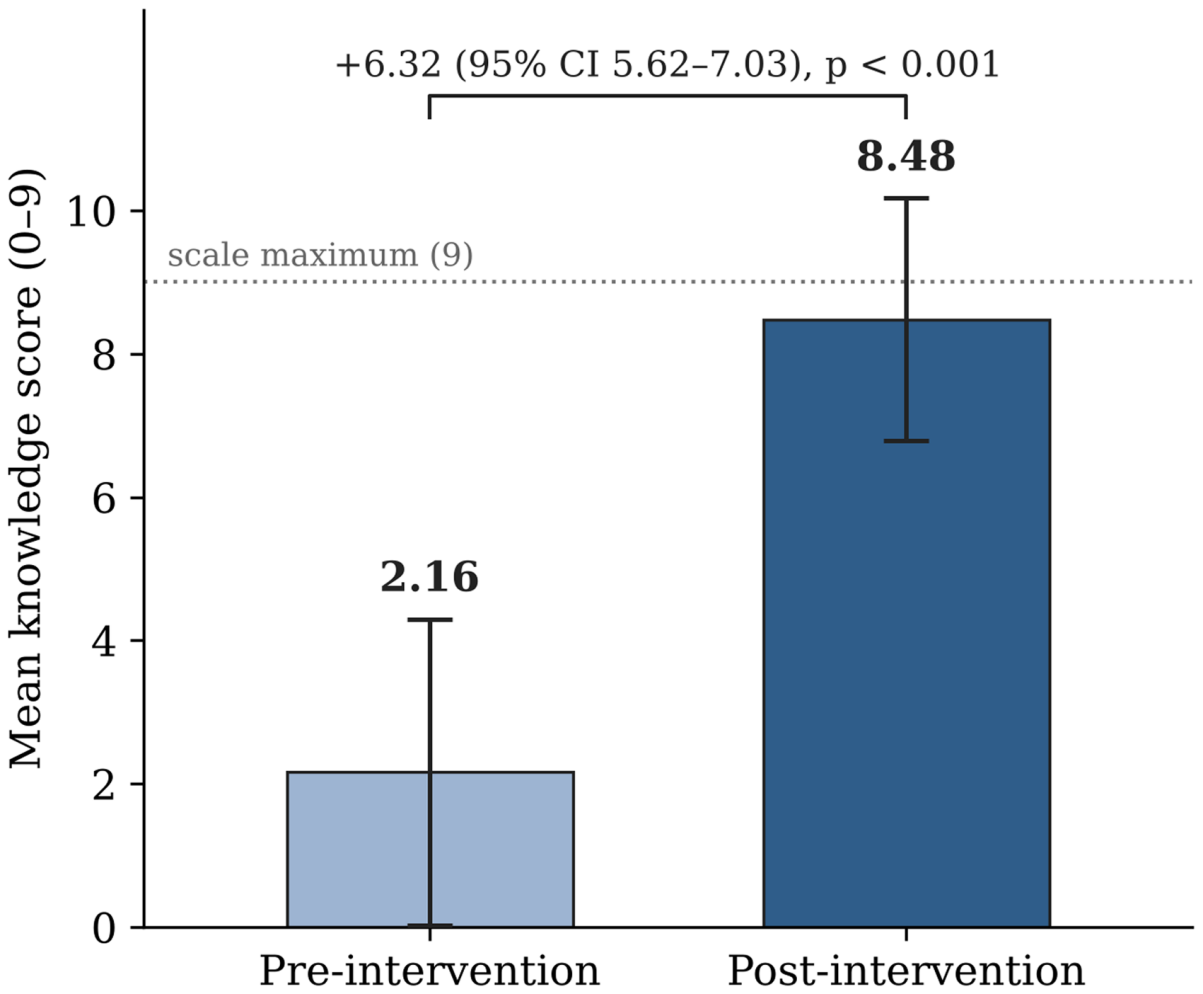
Mean knowledge scores (0–9) before and immediately after the education session in the intervention group (*n* = 81). Error bars represent standard deviations. No corresponding follow-up measurement was made in the control group.

**Table 5:** Knowledge and attitude scores before and after the intervention (intervention group, *n* = 81; paired-samples *t*-test).

Scale (range)	Pre mean ± SD	Post mean ± SD	Mean change (95% CI)	*t* (df)	*p*	*d*
Knowledge (0–9)	2.16 ± 2.14	8.48 ± 1.69	+6.32 (5.62–7.03)	17.84 (80)	<0.001	1.98
Attitude (8–40)	25.79 ± 4.18	35.89 ± 4.81	+10.10 (8.77–11.43)	14.88 (80)	<0.001	1.65

Paired-samples *t*-test, two-sided. Effect sizes are absolute Cohen’s *d* (large ≥ 0.8).

### 3.6 Impact of the intervention on attitude

Attitude scores also improved substantially within the intervention group, from 25.79 ± 4.18 before the intervention to 35.89 ± 4.81 afterward, a mean increase of 10.10 points (95% CI 8.77–11.43); *t*(80) = 14.88, *p* < 0.001, with a large effect size (Cohen’s *d*_z_ ≈ 1.65) (Figure 4; Table 5). After the session, 39 of the 81 women (48.1%) scored 38 or above on the 8–40 scale and 31 scored the maximum of 40, so the distribution approaches the upper limit of the instrument and further change could not have been measured on this scale. Because the control arm was assessed only once, neither of these within-group changes can be compared with a concurrent change in the control arm, and they are reported descriptively, not as an estimate of an intervention effect.

**Figure 4:**
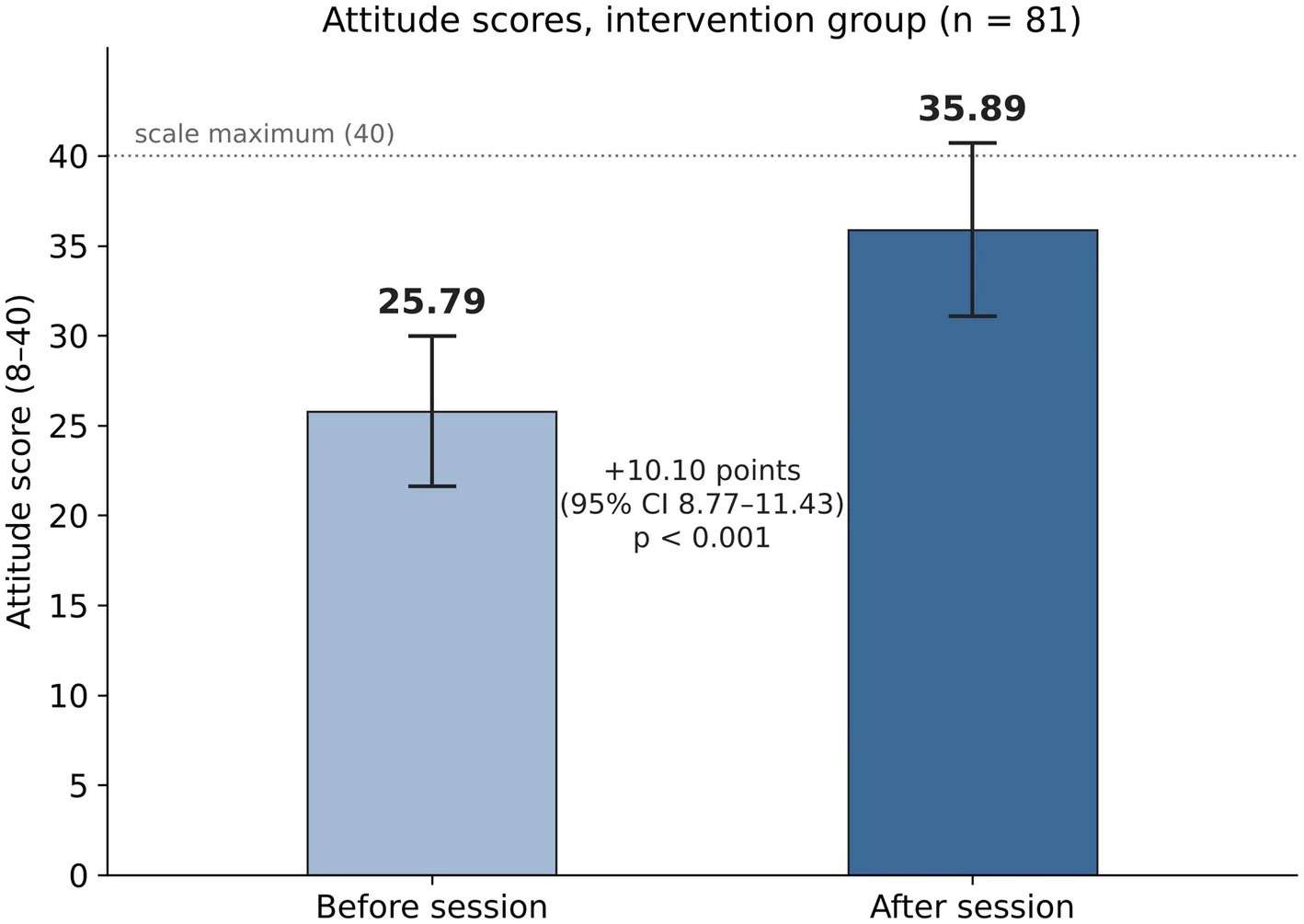
Mean attitude scores (8–40) before and immediately after the education session in the intervention group (*n* = 81). Error bars represent standard deviations. No corresponding follow-up measurement was made in the control group.

### 3.7 Impact of the intervention on postnatal clinic attendance

Postnatal attendance status was available for 169 participants (77 intervention, 92 control); it could not be ascertained for six women (4 intervention, 2 control). Attendance was substantially higher in the intervention arm, where 56 of 77 women (72.7%) attended, than in the control arm, where 38 of 92 (41.3%) did: a difference of 31.4 percentage points (95% CI 17.3–45.6) and a risk ratio of 1.76, so that approximately three women would need to receive the intervention for one additional postnatal consultation to take place (95% CI 2–6). Both arms exceeded the 29% national attendance rate reported for 2019, and the intervention arm more than doubled it (Figure 5). The unadjusted odds ratio was 3.79 (95% CI 1.98–7.27; Pearson *χ*^2^ = 16.77, df = 1, *p* < 0.001; Table 2). Under the four extreme assumptions about the six women without an outcome, the odds ratio ranged from 3.02 (95% CI 1.62–5.64) to 3.86 (2.03–7.34) and remained significant throughout.

**Figure 5:**
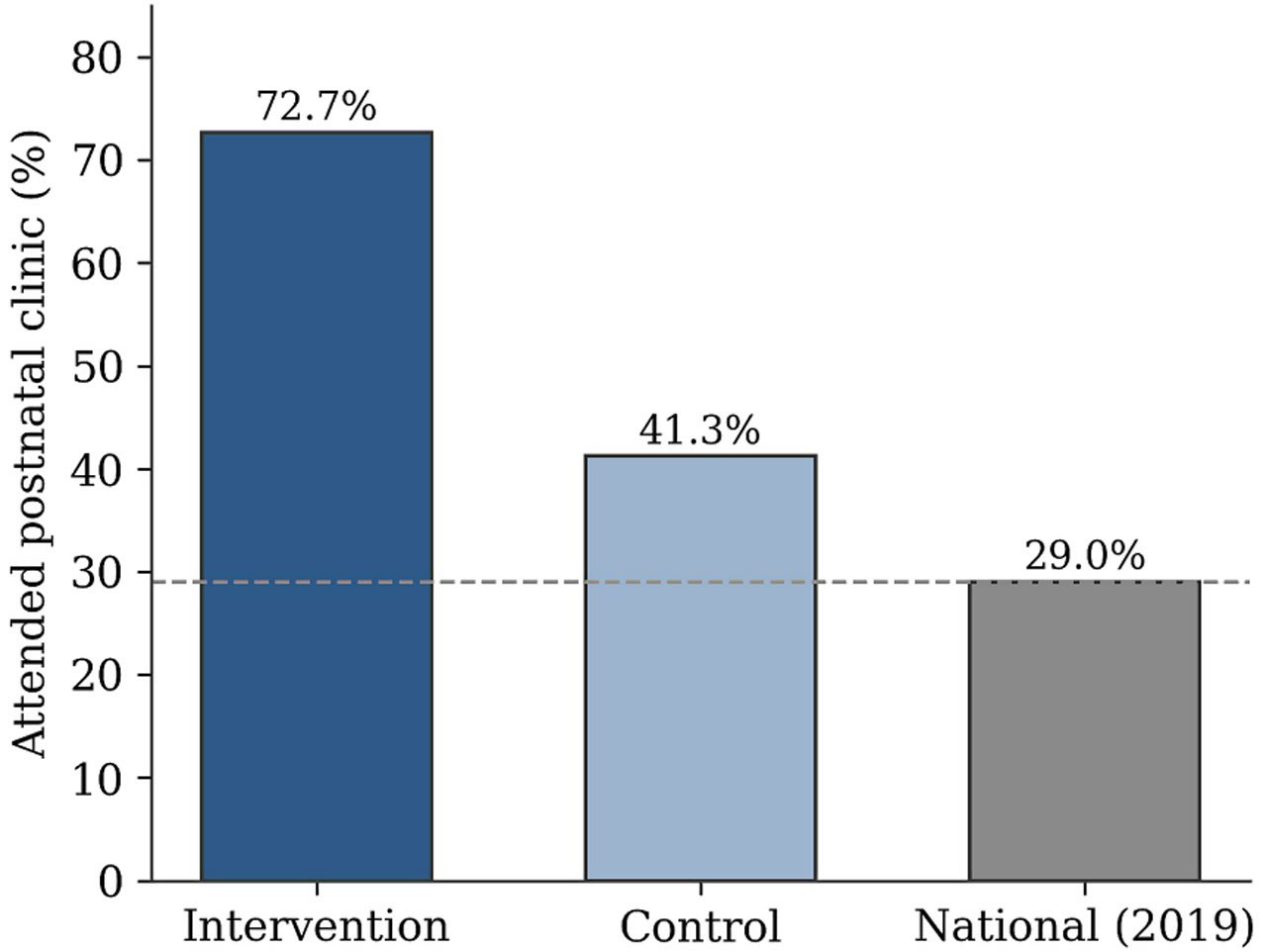
Postnatal clinic attendance by study group, with the 2019 national reference rate (29%; dashed line).

Attendance by individual center qualifies this result (Table 2). In the two control centers it was 13 of 49 (26.5%) and 25 of 43 (58.1%), a difference of 31.6 percentage points, as large as the difference between the study arms; in the two intervention centers it was 23 of 33 (69.7%) and 33 of 44 (75.0%), a difference of 5.3 points. The intraclass correlation for attendance, estimated within arms so as to exclude the intervention effect itself, was 0.091, corresponding to a design effect of 4.73 and an effective sample of approximately 36 women. Applying that design effect to the unadjusted comparison widens the 95% confidence interval for the odds ratio to include the null. With two centers per arm, the difference between arms cannot be separated from variation between centers; the implications are taken up in Section 4.6.

### 3.8 Adjusted analysis (binary logistic regression)

A binary logistic regression estimated the association between the intervention and attendance after adjustment for baseline age, family income, knowledge, and attitude (Table 6; Figure 6). Intervention-group women remained more likely to attend (adjusted OR 5.55, 95% CI 2.04–15.08, *p* = 0.001). The adjusted odds ratio is larger than the unadjusted 3.79, not smaller, so the baseline differences between arms do not account for the association; because knowledge and attitude may lie on the causal pathway from intervention to attendance, however, the adjusted figure is an association conditional on those variables rather than a total effect, and the unadjusted comparison remains the more appropriate summary. None of the covariates was significantly associated with attendance: adjusted odds ratios were 2.12 and 2.16 for the two older age groups, 0.58 and 0.38 for the two higher income categories, 0.58 for high baseline knowledge, and 1.22 for positive baseline attitude, each with a confidence interval spanning 1. The model was fitted to 169 women with 94 events and 75 non-events and estimated seven coefficients, about 13 events and 11 non-events per parameter. Calibration was adequate (Hosmer–Lemeshow *χ*^2^ = 11.71, df = 8, *p* = 0.165), explained variation was modest (Cox and Snell *R*^2^ = 0.132; Nagelkerke *R*^2^ = 0.176), and variance inflation factors ranged from 1.10 to 2.16, indicating no material collinearity.

**Table 6:** Adjusted odds ratios for postnatal clinic attendance (binary logistic regression).

Variable	Category	Adjusted OR	95% CI	*p*-value
Group	Control	1.00 (reference)	–	–
	Intervention	5.55	2.04–15.08	0.001
Age	<25 years	1.00 (reference)	–	–
	25–30 years	2.12	0.82–5.47	0.122
	>30 years	2.16	0.81–5.74	0.124
Income	<500 BD	1.00 (reference)	–	–
	500–1,000 BD	0.58	0.26–1.31	0.191
	>1,000 BD	0.38	0.11–1.29	0.121
Knowledge	Low	1.00 (reference)	–	–
	High	0.58	0.29–1.16	0.124
Attitude	Negative	1.00 (reference)	–	–
	Positive	1.22	0.49–3.02	0.669

Dependent variable: postnatal clinic attendance (attended vs not attended). The intervention group was the only significant predictor (*p* < 0.001).

**Figure 6:**
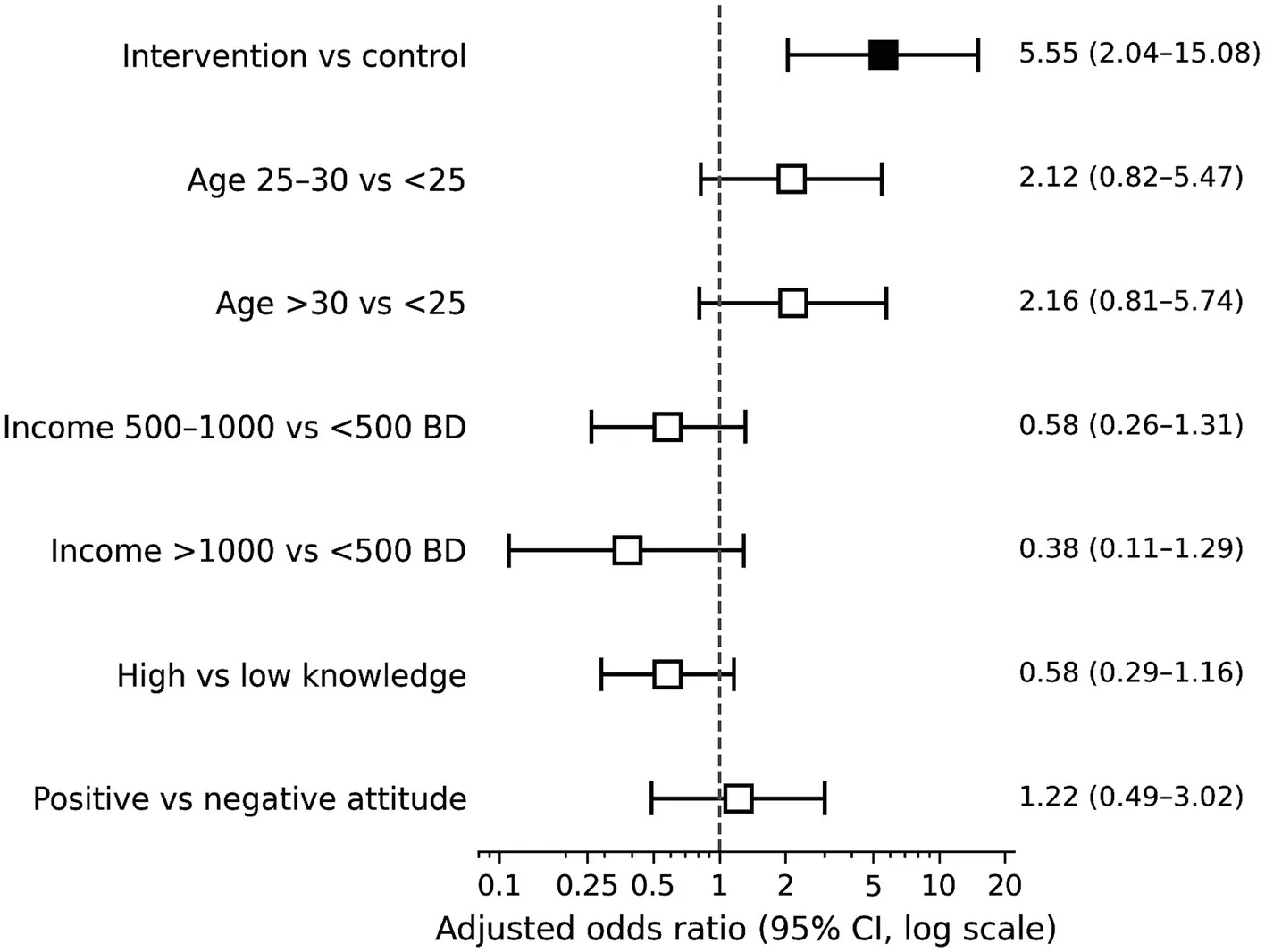
Adjusted odds ratios (squares) with 95% confidence intervals (horizontal lines) for postnatal clinic attendance from the binary logistic regression. The filled square denotes a statistically significant association; the dashed line marks the null value (OR = 1).

### 3.9 Obstetric and medical factors associated with attendance

Across the whole sample, one obstetric or medical factor was significantly associated with attendance: women taking daily medication were less likely to attend than those who were not (34.2% versus 61.8%; *χ*^2^ = 9.10, Fisher’s exact *p* = 0.003). Having previously waited too long before starting a postnatal consultation was not significantly associated with attendance (*n* = 29; Fisher’s exact *p* = 0.092). No other obstetric or medical characteristic was significantly associated with attendance (Table 7). These comparisons were exploratory and were not adjusted for multiple testing.

**Table 7:** Association between obstetric and medical characteristics and postnatal clinic attendance.

Characteristic	Category	Non-attendance *n* (%)	Attendance *n* (%)	Statistics	*p*
Previous pregnancy	No	20 (48.8)	21 (51.2)	0.425^*^	0.589
	Yes	55 (43.0)	73 (57.0)		
Number of deliveries	1	23 (48.9)	24 (51.1)	4.057	0.255
	2	10 (35.7)	18 (64.3)		
	3	7 (29.2)	17 (70.8)		
	≥4	15 (51.7)	14 (48.3)		
Mode of last delivery	Vaginal	49 (40.8)	71 (59.2)	3.573^*^	0.074
	Cesarean	6 (75.0)	2 (25.0)		
Previous postnatal complications	No	52 (42.3)	71 (57.7)	0.616^*^	0.651
	Yes	3 (60.0)	2 (40.0)		
Previous antenatal complications	No	55 (43.7)	71 (56.3)	0.770^*^	1.000
	Yes	0 (0.0)	1 (100.0)		
Current pregnancy planned	No	26 (44.8)	32 (55.2)	0.022^*^	1.000
	Yes	48 (43.6)	62 (56.4)		
Attended any postnatal clinic before	No	41 (41.8)	57 (58.2)	0.219^*^	0.677
	Yes	14 (46.7)	16 (53.3)		
Mode of conception	Normal	74 (44.3)	93 (55.7)	0.026^*^	1.000
	Induced	1 (50.0)	1 (50.0)		
Waited too long before consultation	No	6 (75.0)	2 (25.0)	4.067^*^	0.092
	Yes	7 (33.3)	14 (66.7)		
Daily medication	No	50 (38.2)	81 (61.8)	9.104^*^	0.003
	Yes	25 (65.8)	13 (34.2)		

Values are *n* (%) within each row category; percentages are of the row total. The Statistics column reports the Pearson chi-square statistic; where marked with an asterisk, the *p*-value is the two-sided Fisher’s exact probability and the chi-square statistic is shown for reference only. Fisher’s exact test was applied to every 2 × 2 comparison in this table, including those in which all expected cell counts exceeded five. Daily medication was the only characteristic significantly associated with attendance (*p* = 0.003). Analyses in this table were exploratory and were not adjusted for multiple comparisons.

^*^Two-sided Fisher’s exact test.

### 3.10 Relationship between knowledge/attitude and attendance

Within this study population we found no evidence that measured knowledge or attitude independently predicted subsequent attendance; the analyses were not powered to establish an absence of effect. In the intervention arm, attenders and non-attenders did not differ on knowledge before (*p* = 0.601) or after the session (*p* = 0.749), or on attitude before (25.36 versus 26.52; *p* = 0.282) or after it (35.89 versus 35.29; *p* = 0.629). In the control arm they did not differ on baseline knowledge (*p* = 0.174) or attitude (34.74 versus 33.76; *p* = 0.283). Consistent with the adjusted model, attendance tracked the structural components of the intervention, early booking and standardized counseling, more closely than it tracked individual knowledge or attitude. This is an absence of evidence for an individual knowledge or attitude effect, not evidence that knowledge and attitude are unimportant.

## 4 Discussion

This quasi-experimental study asked whether a structured antenatal intervention combining a standardized health-education session with early postnatal booking was associated with better knowledge, attitude, and postnatal clinic attendance among women approaching term in primary health care centers in the Kingdom of Bahrain. Within the intervention arm, knowledge and attitude both rose substantially between the two assessments. Postnatal clinic attendance was 72.7% in the intervention arm against 41.3% in controls, nearly double, and more than twice the 29% national rate recorded in 2019. The association held after adjustment for the baseline differences in age, family income, knowledge, and attitude, which argues against the more favorable demographic profile of the intervention arm as the explanation. Two features of the design, however, limit how far this can be pressed. Allocation was at the level of the health center and was not randomized, and the two control centers differed from each other in attendance by as much as the arms differed from one another. The findings are therefore best read as an association observed in the participating centers, and the rest of this section is written on that basis.

### 4.1 Impact on knowledge and attitude

Knowledge scores rose markedly (mean increase 6.32 points; *d*_z_ = 1.98), consistent with a growing body of trials showing that brief, structured maternal education reliably improves postnatal-care knowledge. In a two-arm randomized controlled trial at Dhulikhel Hospital, Nepal, Chaudhary et al. found that a short, smartphone-delivered postnatal-care education program increased knowledge scores by an additional 8.07 points relative to usual care among pregnant women using smartphones (63), in a population and delivery format close to our own. A community health worker-led, quasi-experimental education program among pregnant women in Eastern Uganda raised the proportion of women with sufficient maternal and child health knowledge from 19.6 to 80.3% (64). Attitude scores rose by a comparable amount in standardized terms (*d*₂ = 1.65), so the session appears to have shifted both what women knew and how they regarded the service; a controlled antenatal-education trial in Mansoura, Egypt, similarly reported gains in both knowledge and attitude after structured prenatal sessions (65), and the Ugandan program improved attitudes alongside knowledge (64). After the session almost half the intervention arm scored 38 or above out of 40 on the attitude scale, so the instrument could not register further improvement in those women and the measured change may understate the true one. Two further cautions apply. The post-intervention questionnaire was administered minutes after the session, so the knowledge gain in particular may reflect immediate recall rather than durable learning. And without a concurrent second measurement in the control arm, none of these within-group changes can be separated from the effects of repeated exposure to the questionnaire, social desirability, or the antenatal visit itself.

### 4.2 Sources of awareness about postnatal care

Among women aware of the postnatal clinic, most had heard about it informally: friends (20%) and family members (18%) together accounted for 38% of reported sources, against 3% for formal health-education sessions and 7% for social media. Family and peers shape maternal health-seeking in other settings too. In a cross-sectional study in Kathmandu, Nepal, Upadhyay et al. showed that family members, particularly husbands, exerted a substantial influence on whether women used maternal health-care services, and argued that maternal health education should extend beyond the individual woman to her family (66). The channels best suited to accurate and standardized messaging are therefore the ones least used. Strengthening provider-led counseling and digital health promotion would widen the reach of postnatal-care information and make it more consistent.

### 4.3 Impact on postnatal clinic attendance

Attendance was substantially higher in the intervention arm: 72.7% against 41.3%, an unadjusted odds ratio of 3.79 and an adjusted odds ratio of 5.55. Both arms exceeded the 29% national rate for 2019, and the control figure of 41.3% is also well above the 27% found in the 2023 national survey (61). Several explanations are plausible and may operate together. The first is measurement reactivity: control-group women were interviewed with a questionnaire that itself set out the purpose, content, and timing of the postnatal visit, and the act of measurement may have made the visit more salient. The second is that the denominators differ: our participants were engaged with antenatal care at 36 weeks or beyond, had consented to follow-up, owned a smartphone, and were free of the conditions that led to exclusion, whereas the national figure covers all antenatal attendees, including women who disengage before term. The third is that attendance may simply have risen since 2019, a benchmark that predates the return to face-to-face postnatal care after the pandemic and the dissemination of the 2023 survey findings within the Directorate (61). Each of these would act on both arms and would bias the between-group comparison toward the null rather than away from it, so they do not explain the difference we observed, although the first means the control proportion should not be read as the attendance rate of untreated routine care.

What does bear directly on the difference is the variation between centers. Attendance in the two control centers was 26.5 and 58.1%, while the two intervention centers were at 69.7 and 75.0%. One control center therefore performed closer to the intervention centers than to the other control center, and the intraclass correlation of 0.091 reduces the effective sample to about 36 women. With only two centers per arm, an intervention effect and a center effect cannot be separated, and the between-arm difference should be read with that in mind.

The magnitude of the association is nonetheless in line with the wider literature on structured maternal interventions. A 2023 systematic review and meta-analysis by Gayesa et al. found that mHealth interventions increased postnatal-care utilization with a pooled odds ratio of 4.13 (95% CI 1.90–8.97) (51), and Shiferaw et al. reported higher uptake among women receiving an mHealth and provider-training intervention in Ethiopia (50). Trials in which the postnatal visit was scheduled in advance point in the same direction: in a randomized trial among low-income women in the United States, scheduling the postpartum visit before hospital discharge was associated with high attendance in both arms (67), and in a cluster randomized trial in Addis Ababa, enhanced SMS and voice reminders improved postnatal compliance with an adjusted odds ratio of 2.98 (95% CI 1.51–5.8) (68). Our design cannot say which of the three bundled components carried the association, but this literature makes the early booking a plausible candidate.

### 4.4 Knowledge, attitude, and the gap with attendance

Knowledge and attitude moved, but behavior did not follow them in any individual sense. Within this study population we found no evidence that measured knowledge or attitude independently predicted attendance, in the unadjusted comparisons or in the adjusted model, where the odds ratio for positive baseline attitude was 1.22 (95% CI 0.49–3.02). That is not the same as showing that knowledge and attitude do not matter; the study was not designed to establish an absence of effect. What it does suggest is that attendance in this sample depended less on what a woman knew or felt than on the structural components of the intervention: standardized counseling and, above all, a concrete early booking. This fits the intention–behavior gap described in maternal care. In a population-based cohort study in Nyanza Province, Kenya, Creanga et al. found that most women intended to use postnatal care, yet actual utilization reached only 51.8%; access- and delivery-related factors, rather than intention, explained the discordance (69). Converting knowledge and favorable attitudes into attendance therefore requires acting on the service-delivery pathway, not only on what women understand.

### 4.5 Obstetric and medical correlates of attendance

One obstetric or medical factor was associated with attendance in exploratory analyses: women on daily medication were less likely to attend (34.2% versus 61.8%; Fisher’s exact *p* = 0.003). This runs against expectation, since such women are usually more engaged with health services; competing care demands, or management of their conditions elsewhere, may explain it. The subgroup is small and the comparison was one of many, so the observation is offered only as a hypothesis for dedicated study. An apparent association between having previously waited too long before a postnatal consultation and subsequent attendance did not survive the exact test appropriate to its cell counts (*p* = 0.092) and is not discussed further. The regional picture is similar. In a cross-sectional study of postpartum women enrolled in team-based care in Alahsa, Saudi Arabia, Alkhamis et al. found that only 50.8% attended at least one postpartum visit, with the leading reasons for non-attendance being a perception of feeling well and a lack of awareness of the clinic or its importance (70). That awareness- and perception-related barriers persist in a comparable Gulf primary-care setting speaks to the relevance of the standardized counseling in our intervention. The 2023 Bahraini survey found the same pattern locally: women counseled by a provider about the importance of the visit, the services provided, and the booking process were significantly more likely to attend, while childcare responsibilities, a perception that the visit was unimportant, and uncertainty about booking dominated the reasons for staying away (61). Our results take those cross-sectional associations a step further by showing prospectively that acting on the counseling-and-booking pathway does raise attendance.

### 4.6 Strengths and limitations

The study addressed a question of direct policy relevance in a setting where postnatal-care utilization has been persistently low. It used a two-arm comparative design with an objectively recorded behavioral outcome ascertained identically in both arms, applied the knowledge and attitude scales before and after the education session in the intervention arm, reported attendance for each participating center, and adjusted the primary comparison for the baseline differences between groups.

The limitations are substantial and we set them out in the order of their consequence for interpretation.

The most consequential concerns allocation and clustering. The four centers were selected by convenience and assigned to arms without randomization, and the two arms coincide with the two governorates, so any systematic difference between governorates or centers in staffing, catchment population, local practice, or the way appointments are handled is confounded with the intervention. We hold no center-level data on service volume, staffing, or historical attendance with which to demonstrate comparability, and the participant-level differences in age, income, and attitude show that the catchment populations were not equivalent. The center-level results make the consequence concrete: attendance in the two control centers differed by 31.6 percentage points, as much as the arms differed from each other, and one control center attended at a rate closer to the intervention centers than to its own arm. The intraclass correlation of 0.091 reduces the effective sample size to about 36 women, and applying the corresponding design effect widens the confidence interval for the unadjusted odds ratio to include the null. No reanalysis of these data can resolve this; it is a property of a design with two centers per arm, which cannot distinguish an intervention effect from a center effect. The findings should be read as an association observed in the participating centers.

Second, the knowledge and attitude scales were administered twice in the intervention arm but only once, at enrolment, in the control arm. There is therefore no concurrent measurement against which the within-group change can be compared, no difference-in-differences estimate can be computed, and the pre/post findings cannot establish that the session caused the change; repeated exposure to the questionnaire, social desirability, and the antenatal visit itself would all act on the intervention arm alone. Post-intervention scores were also collected minutes after the session and reflect short-term change. The attendance outcome is unaffected, having been ascertained identically in both arms.

Third, the attitude scale contains three negatively worded items whose scores had not been reversed in the original analysis. This was identified during revision of the manuscript and has been corrected throughout; all attitude results reported here use the corrected scoring. The correction is not small: the baseline difference between arms is 8.33 points rather than 2.17, the within-group change in the intervention arm is 10.10 points rather than 3.26, and the adjusted odds ratio for the intervention is 5.55 rather than 6.20. That an instrument developed for this study and not previously validated was scored incorrectly at first pass is itself a reason for caution; the corrected scale has good internal consistency, but formal validation remains to be done.

Fourth, two alternative explanations for the between-arm difference cannot be excluded. Measurement reactivity is the more likely: control-group women were interviewed with a questionnaire that itself described the postnatal visit, which may have raised its salience in the comparison arm. Contamination is less likely, since the arms lie in different governorates with separate catchment populations and no shared clinical staff, but midwives across the primary health care system meet in training and professional forums and diffusion of the counseling approach cannot be ruled out. Both would narrow the difference rather than widen it, so if either operated the reported difference is an underestimate; but the control proportion of 41.3% should not be taken as the attendance rate of untreated routine care.

Fifth, the achieved sample fell short of the target. The calculation, based on a one-sided test of two proportions, required 83 per arm and set a target of 100 per arm with allowance for attrition; 175 women were enrolled, leaving the intervention arm two below the minimum. The calculation assumed independent observations and did not account for clustering, and all reported tests were two-sided, so the achieved power was lower than the nominal figure implies, particularly for the secondary and subgroup analyses. Attendance could not be ascertained for six women, although the result was insensitive to how they were handled.

Sixth, several features limit generalizability. Smartphone ownership was required in both arms, which preserved comparability but excludes women who may face the greatest barriers to care. The centers were selected partly for having nurse-midwives already trained in health education, so the intervention was delivered where the capacity to deliver it already existed. Attendance was ascertained from electronic records at the woman’s own center, so visits made elsewhere or recorded outside the mother-and-child health module would have been missed, equally in both arms. Fidelity of delivery was not monitored. The exploratory analyses of obstetric and medical correlates, of the knowledge–attitude–attendance relationship, and of sources of awareness involved many comparisons in small subgroups without adjustment for multiplicity and are hypothesis-generating only. And the study was conducted among Bahraini women and non-Bahraini women married to Bahrainis in four selected centers, which limits transfer to other populations and settings.

### 4.7 Implications for practice and policy

A brief, standardized antenatal counseling session paired with a concrete early booking is delivered within an existing antenatal visit, and attendance in the centers that offered it was substantially higher than in those that did not. We did not evaluate implementation cost, staff time, provider workload, counseling fidelity, acceptability, or sustainability, so scalability was not demonstrated; this is best described as a potentially low-cost intervention that could be integrated into routine antenatal care, subject to evaluation of implementation feasibility, resource requirements, and sustainability. The findings are consistent with a beneficial effect of the intervention, although residual confounding and center-level differences cannot be excluded.

What the study contributes is narrower and, we think, more useful than an effect estimate. Attendance moved while measured knowledge and attitude did not predict it, which suggests that education coupled with a concrete service-delivery mechanism achieves more than information provision alone. Primary-care services that want to raise postnatal-care uptake should build routine early booking and provider-led counseling into the antenatal pathway rather than rely on information alone. Awareness currently travels mainly through informal social networks while formal and digital channels are under-used, so standardizing provider counseling and developing trusted digital materials would extend accurate messaging and reinforce the booking process. Because the intervention bundled a trained-midwife counseling session, an advance appointment, and QR-coded material, this study cannot say which component carried the association; a factorial or stepped design would be needed to separate them.

## 5 Conclusion

In this non-randomized quasi-experimental study in Bahraini primary health care, a brief, standardized antenatal education session combined with early postnatal booking was associated with substantially higher postnatal clinic attendance, an association that held after adjustment for baseline differences. Within the intervention arm, knowledge and attitude both improved between the two assessments, although without a second measurement in the control arm these changes cannot be attributed to the session. Attendance tracked the structural components of the intervention, standardized counseling and a concrete early booking, rather than individual knowledge or attitude. These findings must be read against the variation between centers: attendance in the two control centers differed as much as attendance between the arms, so the design cannot distinguish an effect of the intervention from an effect of the centers in which it was delivered. Embedding routine early booking and provider-led counseling into antenatal care may be a feasible strategy for improving postnatal-care utilization. Larger multicenter studies, preferably cluster-randomized or using stronger quasi-experimental designs, are needed to confirm the effect, assess sustainability, determine implementation costs, and identify which component of the intervention matters.

## Data Availability

The raw data supporting the conclusions of this article will be made available by the authors, without undue reservation.
