# Supplementary material for: Antenatal health education with early postnatal booking and postnatal clinic attendance in primary health care centers in Bahrain: a quasi-experimental study
Source: Front Public Health. 2026 Oct 20;14:1938634. doi: 10.3389/fpubh.2026.1938634 (PMC13727573; doi:10.3389/fpubh.2026.1938634)
Supplement: Supplementary file 1 [file Supplementary_file_1.pdf]

Supplementary Material

Antenatal health education with early postnatal booking and postnatal clinic attendance in primary health care centers in Bahrain: a quasi-experimental study

Alhaddad MEE, Hubail MAH, Rashed MMA, Alnajjar IBS, Husain TKS, AlAali FA, Al-Asoomi H, Makarem B, Chlif S, Habbash F, Almarabheh A, Ben Salah A, Shehata MH. Frontiers in Public Health (2026).

This file contains the standard operating procedures (SOPs) used in the study, as written by the study team in January 2024 before data collection began. SOP 1 governed the intervention centers; Tasks 6 and 7 of SOP 1 constitute the early-booking procedure referred to in Section 2.6 of the article. SOP 2 governed recruitment and data collection in the control centers and is included so that the two pathways can be compared. The procedures are reproduced as written, with spelling and formatting standardized; no steps have been added or removed.

Abbreviations: ATT, attitude score; CPR, Central Population Register (national identification) number; EDD, expected date of delivery; HE, health education; I-SEHA, the electronic health record system of the Bahrain primary health care centers; KN, knowledge score; LMP, last menstrual period; MCH, mother and child health; MOH, Ministry of Health; QIP, quality-improvement project; SD, sociodemographic data.

SOP 1. Recruitment, health education, early postnatal booking and data collection in the intervention centers

**SOP number**	1
**Prepared by**	Clinical Master in Family Medicine (CMFM) Cohort 3 study team
**Date**	January 2024
**Scope**	Describes recruitment, the health-education session, early booking of the postnatal appointment, and data collection before and after the intervention in the two intervention centers. Written for the quality-improvement project on postnatal visits; may later be used in a wider context.
**Purpose**	To help the staff involved (MCH clerks, interviewers, midwives) manage the intervention group in a standardized, efficient way with high-quality outputs.
**Prerequisite**	Protocol of the quality-improvement project.

Responsibilities

No.	Role	Responsibilities
1	**MCH clerk**	Produce the patient list; confirm the current appointment; register the postnatal appointment.
2	**Interviewer A**	Pre-intervention interview (SD, KN1, ATT1); complete the relevant questionnaire sections.
3	**Midwife (trained)**	Deliver the HE session; provide the HE material and QR code; give the postnatal appointment date.
4	**Interviewer B**	Verify the appointment date; post-intervention interview (KN2, ATT2); complete the relevant questionnaire sections.
5	**Recruitment responsible**	Select patients from the list; check inclusion and exclusion criteria; explain the project; obtain informed consent.
6	**Postnatal visit follower**	Follow up attendance of enrolled women; record the visit in the questionnaire.

Figure S1 shows the sequence of tasks; each task is described in detail below.

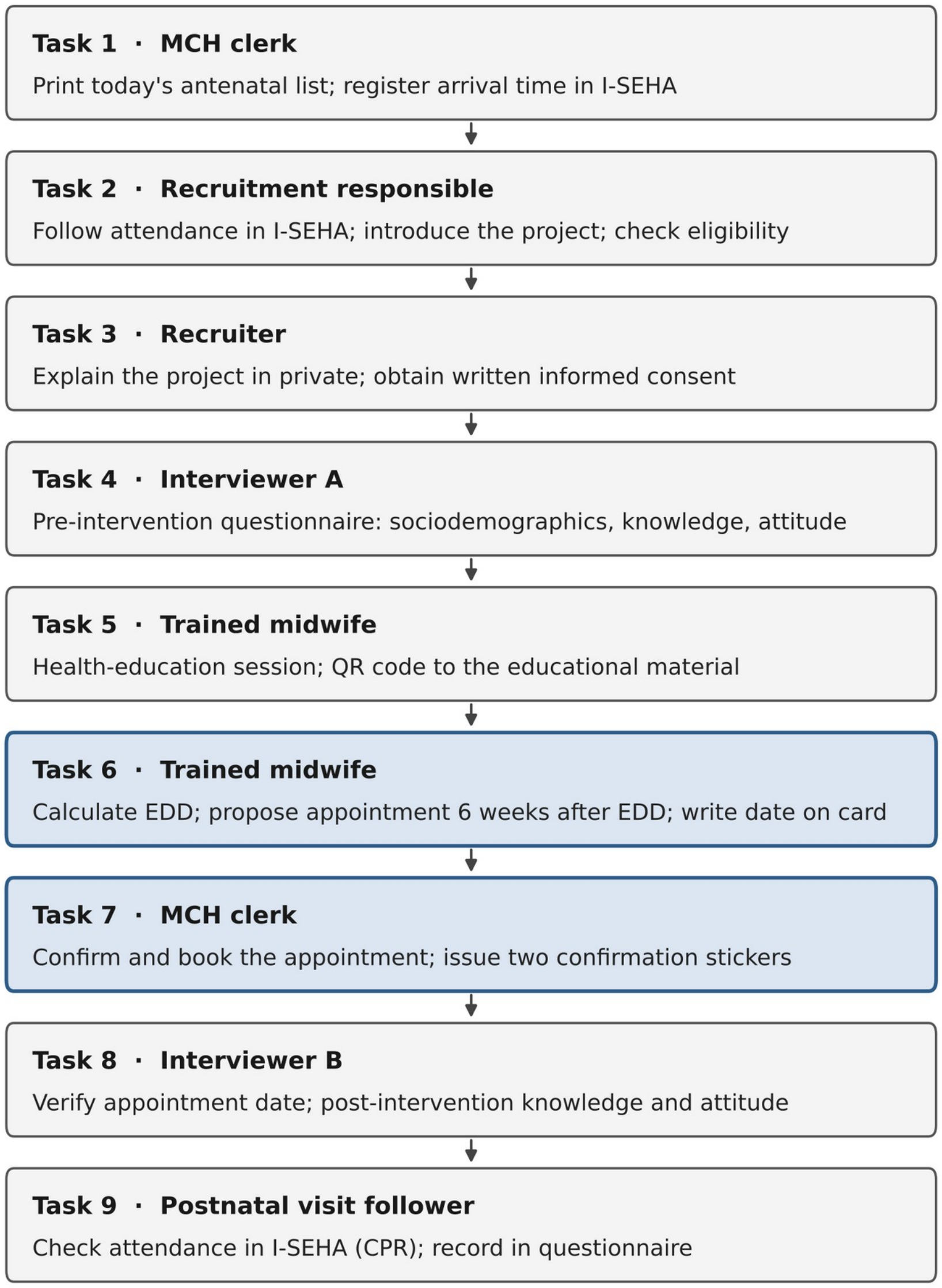

Figure S1. Sequence of tasks in SOP 1. Shaded boxes (Tasks 6 and 7) form the early-booking procedure. Drawn from the task descriptions in this SOP.

Task 1: Patient list production *Role: MCH clerk*

Step	Action	Comments
1	Print the list of antenatal patients for the day.	
2	Register the time of arrival in I-SEHA when the patient arrives at the center.	

Notes: Authorization of the MOH required.

Task 2: Verification *Role: Recruitment responsible*

Step	Action	Comments
1	Take the antenatal patient list from the MCH clerk.	
2	Follow the attendance of antenatal patients through I-SEHA.	
3	Introduce himself or herself and the project to each patient on arrival.	
4	Verify whether the patient meets the inclusion and exclusion criteria.	
5	Patients who do not meet the criteria are not enrolled.	

Task 3: Informed consent *Role: Recruiter*

Step	Action	Comments
1	Give the patient a detailed explanation of the project in a consultation room, for privacy.	Explain the informed consent document.
2	If she is willing to take part, the patient signs the consent form and is guided to the interview room.	
3	If not, the patient is not enrolled.	

Notes: Prepare copies of the written informed consent form; Ensure the interview room is available.

Task 4: Pre-intervention data collection *Role: Interviewer A*

Step	Action	Comments
1	Record sociodemographic data in the appropriate section of the questionnaire.	
2	Record pre-intervention knowledge and attitude in the appropriate sections.	
3	Guide the patient to the midwife's room.	

Notes: Ensure questionnaires are available; Ensure tables, chairs, pens and other materials are available.

Task 5: Health-education session *Role: Midwife*

Step	Action	Comments
1	Deliver the health-education session.	
2	Provide the QR code so the patient can open the session material on her smartphone.	
3	Give the patient time to read and understand the educational material.	
4	Check that the patient has understood the material and answer any questions.	

Notes: Ensure the educational material is available (PDF on the importance of postnatal clinic attendance, hosted online with a URL and QR code); Ensure the midwife is trained to deliver the session using the material; Ensure the midwife knows how to use the QR code.

Task 6: Give the postnatal appointment date *Role: Midwife*

Step	Action	Comments
1	Calculate the expected date of delivery from the last menstrual period or ultrasound.	
2	Set the appointment 6 weeks after the expected date of delivery.	
3	Write the calculated date on a card and give it to the patient.	
4	Direct the patient to the MCH clerk for confirmation.	

Notes: Produce an appointment card showing the health center, patient name, ID number, proposed date and midwife's signature.

Task 7: Confirm the postnatal appointment *Role: MCH clerk*

Step	Action	Comments
1	The patient takes the card to the clerk.	
2	The clerk confirms and books the appointment.	
3	The clerk gives the patient a sticker confirming the appointment.	Two stickers: one for the patient, one for Interviewer B.
4	The clerk guides the patient to the interview room.	

Notes: Ensure stickers are available; Explain the process to the MCH clerks.

Task 8: Post-intervention data collection *Role: Interviewer B*

Step	Action	Comments
1	Verify the postnatal appointment date.	
2	Place the sticker on the appointment list.	Appointment list prepared with full name, ID number and a space for the sticker.
3	Write the appointment date in the questionnaire.	
4	Record post-intervention knowledge and attitude in the appropriate sections.	
5	Inform the patient that her part in the project is complete and thank her.	

Notes: Ensure the questionnaire pre-filled by Interviewer A is available; Ensure tables, chairs, pens and other materials are available; Assign Interviewers A and B to the same person.

Task 9: Follow postnatal attendance and record it in the questionnaire *Role: Postnatal visit follower*

Step	Action	Comments
1	Review the appointment list after one month.	Search I-SEHA by the patient's CPR number; agree the optimal interval with stakeholders.
2	Check whether the patient attended the booked appointment.	
3	If she attended, mark attendance on the appointment list and update the questionnaire.	
4	If she did not attend, check again the following month.	

Notes: Appointment list available; Access to I-SEHA; Questionnaires available.

Reference documents: patient list; health-education material; QR code details; questionnaire.

SOP 2. Recruitment and data collection in the control centers

**SOP number**	2
**Prepared by**	CMFM Cohort 3 study team
**Date**	January 2024
**Scope**	Describes recruitment and data collection in the two control centers. Women in these centers received standard care; no health-education session was delivered and no postnatal appointment was booked by the study.
**Purpose**	To help the staff involved (MCH clerks, interviewers) manage the control group in a standardized, efficient way with high-quality outputs.
**Prerequisite**	Protocol of the quality-improvement project.

Responsibilities

No.	Role	Responsibilities
1	**MCH clerk**	Produce the patient list.
2	**Interviewer**	Interview (SD, KN, ATT); complete the relevant questionnaire sections.
3	**Recruitment responsible**	Select patients from the list; check inclusion and exclusion criteria; explain the project; obtain informed consent.
4	**Postnatal visit follower**	Follow up attendance of enrolled women; record the visit in the questionnaire.

Task 1: Patient list production *Role: MCH clerk*

Step	Action	Comments
1	Print the list of antenatal patients for the day.	
2	Register the time of arrival in I-SEHA when the patient arrives at the center.	

Notes: Authorization of the MOH required.

Task 2: Verification of inclusion and exclusion criteria *Role: Recruitment responsible*

Step	Action	Comments
1	Take the antenatal patient list from the MCH clerk.	
2	Follow the attendance of antenatal patients through I-SEHA.	
3	Introduce himself or herself and the project to each patient on arrival.	
4	Verify whether the patient meets the inclusion and exclusion criteria.	
5	Patients who do not meet the criteria are not enrolled.	

Task 3: Informed consent *Role: Recruiter*

Step	Action	Comments
1	Give the patient a detailed explanation of the project in a consultation room, for privacy.	Explain the informed consent document.
2	If she is willing to take part, the patient signs the consent form and is guided to the interview room.	
3	If not, the patient is not enrolled.	

Notes: Prepare copies of the written informed consent form; Ensure the interview room is available.

Task 4: Data collection *Role: Interviewer*

Step	Action	Comments
1	Record sociodemographic data in the appropriate section of the questionnaire.	
2	Record knowledge and attitude in the appropriate sections.	

Notes: Ensure questionnaires are available; Ensure tables, chairs, pens and other materials are available.

Task 5: Follow-up of postnatal attendance *Role: Postnatal visit follower*

Step	Action	Comments
1	Search I-SEHA by the patient's CPR number.	
2	Check whether the patient booked and attended her postnatal appointment.	
3	Record attendance or non-attendance in the questionnaire.	
4	Compile a report at the end.	

Reference documents: patient list; questionnaire.

Note on outcome ascertainment. Task 9 of SOP 1 and Task 5 of SOP 2 describe the planned follow-up. Postnatal attendance for the analysis was ascertained identically in both arms by review of the electronic record after the 6-month window had closed for the last woman enrolled, as described in Section 2.5 of the article.
